# The CD94/NKG2A inhibitory receptor educates uterine NK cells to optimize pregnancy outcomes in humans and mice

**DOI:** 10.1016/j.immuni.2021.03.021

**Published:** 2021-06-08

**Authors:** Norman Shreeve, Delphine Depierreux, Delia Hawkes, James A. Traherne, Ulla Sovio, Oisin Huhn, Jyothi Jayaraman, Amir Horowitz, Hormas Ghadially, John R.B. Perry, Ashley Moffett, John G. Sled, Andrew M. Sharkey, Francesco Colucci

**Affiliations:** 1 Department of Obstetrics & Gynaecology, University of Cambridge, National Institute for Health Research Cambridge Biomedical Research Centre, Cambridge CB2 0SW, UK; 2 University of Cambridge Centre for Trophoblast Research, Cambridge, UK; 3 Department of Pathology, University of Cambridge, Cambridge, UK; 4 AstraZeneca, Granta Park, Cambridge CB21 6GH, UK; 5 Department of Physiology, Development and Neurobiology, University of Cambridge, Cambridge, UK; 6 Department of Oncological Sciences, Precision Immunology Institute and Tisch Cancer Institute, Icahn School of Medicine at Mount Sinai, New York, NY, USA; 7 MRC Epidemiology Unit, University of Cambridge, Cambridge UK; 8 Department of Medical Biophysics, University of Toronto, Toronto, Canada; 9 Translational Medicine, Hospital for Sick Children, Toronto, Canada

**Keywords:** NK cells, inhibitory receptors, NKG2A, HLA-E, HLA-B, pregnancy, uterine natural killer cells, asymmetric fetal growth, placenta

## Abstract

The conserved CD94/NKG2A inhibitory receptor is expressed by nearly all human and ∼50% of mouse uterine natural killer (uNK) cells. Binding human HLA-E and mouse Qa-1, NKG2A drives NK cell education, a process of unknown physiological importance influenced by *HLA-B* alleles. Here, we show that NKG2A genetic ablation in dams mated with wild-type males caused suboptimal maternal vascular responses in pregnancy, accompanied by perturbed placental gene expression, reduced fetal weight, greater rates of smaller fetuses with asymmetric growth, and abnormal brain development. These are features of the human syndrome pre-eclampsia. In a genome-wide association study of 7,219 pre-eclampsia cases, we found a 7% greater relative risk associated with the maternal *HLA-B* allele that does not favor NKG2A education. These results show that the maternal HLA-B→HLA-E→NKG2A pathway contributes to healthy pregnancy and may have repercussions on offspring health, thus establishing the physiological relevance for NK cell education.

**Video Abstract:**

## Highlights

• CD94/NKG2A educates uterine NK cells
• NKG2A-deficient dams display reduced utero-placental hemodynamic adaptations
• Asymmetric growth restriction and abnormal brain development in NKG2A-deficient dams
• Non-functional HLA-B→HLA-E→NKG2A pathway exposes women to greater pre-eclampsia risk

## Graphical abstract

**Figure:**
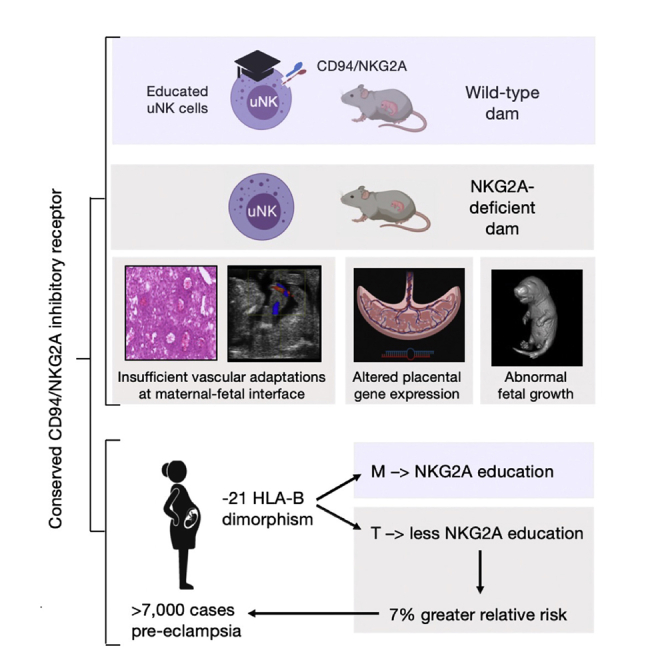

## Teaser

The physiological importance of NK cell education is unclear. Shreeve et al. show that the CD94/NKG2A receptor educates maternal NK cells to orchestrate vascular changes leading to normal fetal brain development and, in humans, to lower risk of complicated pregnancy.

## Introduction

Inhibitory receptors are vital checkpoints in the immune system. In addition to suppressing activation, inhibitory natural killer (NK) cell receptors prime and calibrate NK cell function, a phenomenon known as NK cell education (Orr and Lanier, 2010) or licensing (Kim et al., 2005). Key inhibitory receptors able to educate NK cells are the conserved and invariable c-type lectin CD94/NKG2A inhibitory receptor (hereafter called NKG2A, which stands for Natural Killer cell protein Group 2-A) that binds non-classical human leukocyte antigen (HLA)-E (Le Luduec et al., 2019; Björkström et al., 2010; Brodin et al., 2009; Yawata et al., 2008; Anfossi et al., 2006; Kim et al., 2005) and the polymorphic killer-cell immunoglobulin-like receptors (KIR) that bind classical HLA class I molecules HLA-A, HLA-B, and HLA-C. HLA-E requires the supply of peptides from classical HLA-A, HLA-B, or HLA-C for appropriate folding and transport to the cell surface (Braud et al., 1998a, 1998b; Lee et al., 1998). There is a dimorphism at the −21 position of the leader sequence supplied by HLA-B (−21 HLA-B) encoding either threonine (T) or methionine (M) (Yunis et al., 2007; Valés-Gómez et al., 1999). This separates individuals into those who can provide functional peptides for high HLA-E expression and NKG2A ligation, which leads to education (MT or MM), and those who cannot (TT) and therefore have low HLA-E expression (Horowitz et al., 2016). NKG2A-driven education in MT or MM individuals results in phenotypically more diverse NK cell populations with increased functional potency (Horowitz et al., 2016). The two alleles *A* and *G* of the single-nucleotide polymorphism (SNP) rs1050458 encode the two isoforms of the HLA-B leader peptide −21M and −21T, respectively. A role for this *HLA-B* dimorphism is emerging in HIV control, immunotherapy of patients with leukemia, and graft versus host disease (Ramsuran et al., 2018; Hallner et al., 2019; Petersdorf et al., 2020).

Although the mechanisms underlying NK cell education are becoming clear (Goodridge et al., 2019), and NKG2A-educated NK cells have enhanced responses and are metabolically more resilient than KIR-educated NK cells (Highton et al., 2020), how NKG2A education affects physiology is unclear (Boudreau and Hsu, 2018). Although peripheral blood NK cells are ∼50% NKG2A^+^, a specialized population of uterine NK (uNK) cells that contribute to reproduction by regulating maternal vascular remodeling and early placentation (Moffett and Shreeve, 2015) are ∼95% NKG2A^+^ (Björkström et al., 2016). Inhibitory uNK cell receptors are regulated in a tissue-specific manner by maternal self-HLA molecules (Sharkey et al., 2015) in steady state, and during pregnancy, NKG2A^+^ uNK cells can bind fetal HLA-E expressed by invading extravillous trophoblast (EVT) (King et al., 2000). There is no evidence that fetal HLA-C, HLA-E, or HLA-G educates human uNK cells, and in the mouse, it is clear that maternal, not fetal, major histocompatibility complex (MHC) educates uNK cells (Kieckbusch et al., 2014). Fetal HLA-C, for example, by interacting with strongly inhibitory KIR2DL1, may lead to low birth weight and increased pre-eclampsia risk, probably because this interaction suppresses activation rather than education of uNK cells (Hiby et al., 2004, 2010, 2014). How the maternal HLA-B→HLA-E→NKG2A pathway affects the outcome of pregnancy is unknown.

Pre-eclampsia is a systemic syndrome that affects ∼5% of all pregnancies and can be associated with fetal growth restriction (FGR) (Mol et al., 2016). Characterized by a range of features, including recent-onset hypertension and proteinuria, pre-eclampsia is a leading cause of maternal and perinatal morbidity and mortality. Although the pathophysiology of pre-eclampsia is multi-factorial, abnormal placental development, altered placental perfusion, and endoplasmic reticulum stress in early pregnancy are likely to underpin most cases (Burton et al., 2019). Both maternal and fetal genomes contribute to disease risk (Skjaerven et al., 2005). Candidate gene approaches in case-control and genome-wide association studies (GWASs) have revealed associations with genes involved in blood pressure regulation, immune responses, lipid metabolism, and coagulation, consistent with the multifactorial and polygenic nature of the disorder. A role for the immune system and uNK cells in the pathogenesis of pre-eclampsia and the regulation of placentation and fetal growth is also likely (Redman and Sargent, 2005; Colucci, 2019; Moffett and Colucci, 2014). Epidemiological evidence in Europeans and reproduced in a sub-Saharan population (Hiby et al., 2004, 2010; Kennedy et al., 2016; Nakimuli et al., 2015) gives rise to our working hypothesis that inhibitory combinations of maternal *KIR* and fetal *HLA-C* alleles impede placentation through excessive uNK cell inhibition, whereas a background of uNK cell activation associates with lower pre-eclampsia risk and might promote higher birthweight (Moffett and Colucci, 2015; Hiby et al., 2014). This hypothesis is supported by our studies in mice, in which some aspects of the pathology, such as FGR and insufficient vascular remodeling, are recapitulated when uNK cells are strongly inhibited (Kieckbusch et al., 2014). A reduction in human birthweight, even as small as 5%–10%, is clinically relevant (Juárez and Merlo, 2013), and low birthweight predisposes individuals to hypertension and diabetes in adulthood (Knop et al., 2018). The magnitude of FGR caused by hypofunctional or absent NK cells in mice is in the same 5%–10% range (Kieckbusch et al., 2014).

The ligand for mouse NKG2A is Qa-1, and like HLA-E, its expression depends on peptides derived from classical MHC class I molecules. The expression of Qa-1 and other non-classical MHC molecules appears negligible on mouse trophoblast cells (Madeja et al., 2011); thus, the mouse offers the opportunity to assess the role of NKG2A-mediated uNK cell education by maternal self-MHC, eliminating potential confounding effects introduced by fetal MHC. Here we show that NKG2A^+^ uNK cells are functionally more responsive than NKG2A^−^ uNK cells in wild-type (WT) mice. By comparing WT and NKG2A-deficient *Klrc1*^−/−^ dams, we define a specific role of NKG2A in maternal vascular adaptation to pregnancy, fetal growth, and brain development. Finally, we show that pre-eclampsia is less prevalent in women carrying the −21M HLA-B genotype that favors NKG2A education, suggesting that the maternal HLA-B→HLA-E→NKG2A pathway optimizes pregnancy outcome.

## Results

### NKG2A educates mouse uNK cells

**Figure 1:**
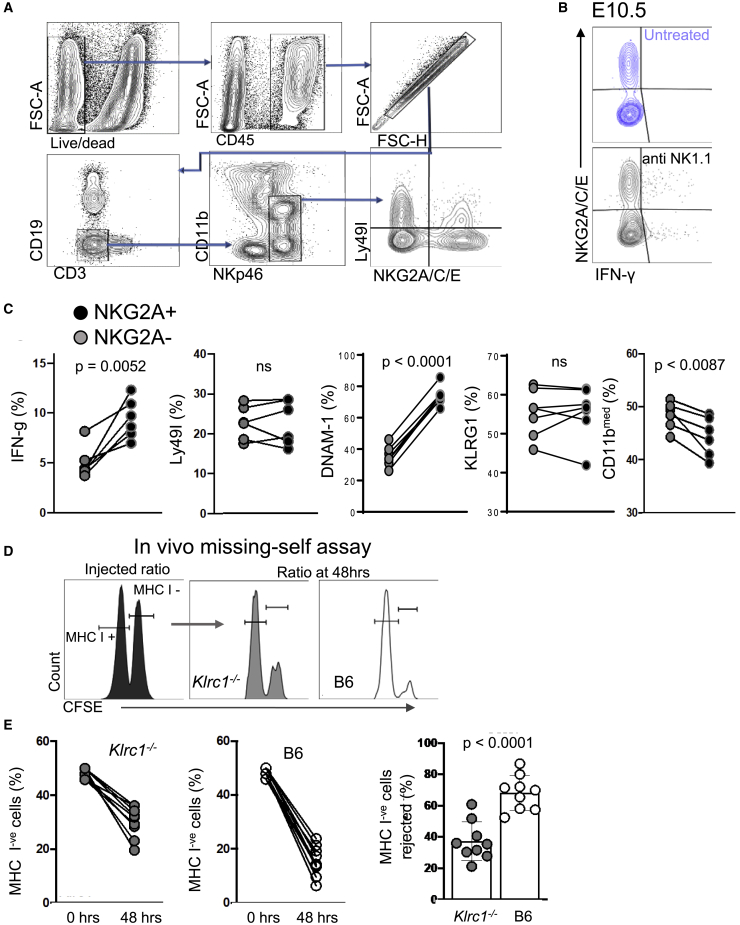
NKG2A educates uterine NK cells and regulates missing-self response (A and B) Representative (from 2 experimental repeats) flow cytometric gating strategy (A) for education assay (percentage of responding cells) on E10.5 uNK cells and (B) for intracellular IFN-γ in uNK cells untreated or activated by anti-NK1.1 crosslinking. (C) Functional and phenotypic characterization of NKG2A+ (black dots) and NKG2A− uNK cells (gray dots). Shown are proportions of cells staining positive for intracellular IFN-γ upon anti-NK1.1 crosslinking or for cell-surface markers Ly49I, DNAM-1, KLRG1, and CD11b in B6 mice (n = 6, each datapoint represents 1 mouse). (D) Representative histograms (from 2 experimental repeats) of flow cytometric analysis for missing-self assay showing the ratios of carboxyfluorescein diacetate succinimidyl ester (CFSE)-labeled B6 (MHC-I^+^) over *B2m*^−/−^ (MHC-I^−^) splenocytes at the time of injection and after 48 h in syngeneic NKG2A-deficient *Klrc1*^−/−^ and B6 mice. (E) Quantification of MHC-I^+^ over MHC-I^−^ cell ratios in the spleen of individual *Klrc1*^−/−^ and B6 host mice (n = 9, each datapoint represents 1 mouse), and in the right panel, quantification of the percentages of rejected MHC-I^−^ cells by *Klrc1*^−/−^ and B6 host mice. Paired t tests and t tests were used in (C) and in (E), respectively. Error bars in (E) represent standard deviation. See also Figure S1.

Most murine and human peripheral NK cells display a bi-modal expression pattern for NKG2A. We show here that both splenic and uterine mouse NK cells expressed NKG2A but neither expressed NKG2C or NKG2E at embryonic day (E) 9.5, because cells from NKG2A-deficient *Klrc1*^−/−^ mice on a C57BL/6 (B6) background did not stain with an antibody that reacts with NKG2A, NKG2C, and NKG2E (Figure S1A), consistent with published data (Rapaport et al., 2015). NKG2A can also be expressed by activated CD8^+^ T cells, but at E9.5, there was no expression of NKG2A on T cells in the uterus or the spleen of WT B6 mice (Figure S1B). Therefore, in B6 mice, NKG2A is solely expressed on NK cells and not accompanied by other NKG2 receptors. In the early-gestation mouse uterus, there are three subsets of innate lymphoid cells (ILCs): conventional NK cells (cNKs), uterine ILC1 (uILC1), and tissue-resident uNK (trNK) cells (Doisne et al., 2015; Filipovic et al., 2018). We confirmed that all three subsets express NKG2A in early gestation, from ∼40% in cNK and trNK to ∼60% in uILC1 (Figure S1C). For simplicity, in this paper, we refer to these three subsets collectively as uNK cells. Both the NKG2A^+^ and NKG2A^−^ uNK subsets expressed the cell-surface markers of education (DNAM-1) and maturation (KLRG1) (Figure S1D). The NKG2A ligand Qa-1 (the mouse ortholog of HLA-E) is not found on mouse trophoblast (Madeja et al., 2011), and we show here that it was expressed by uterine CD45^+^ leucocytes, including T lymphocytes, B lymphocytes, and NK cells, in early pregnancy (Figures S1E−S1F). In mice, interferon gamma (IFN-γ) produced by NK cells is the key cytokine involved in remodeling the uterine spiral arteries (Ashkar et al., 2000), a necessary step for optimal placentation. Production of IFN-γ through stimulation of activating receptors is also a standard functional readout of both human and mouse NK cell education. To quantify the contribution of NKG2A to uNK cell education in mice, we gated on all uNK cells and compared the percentage of IFN-γ^+^ cells upon NK1.1 stimulation within NKG2A^+^ and NKG2A^−^ subsets in B6 dams (Figures 1A and 1B). This was greater within NKG2A^+^ uNK cells than within NKG2A^−^ uNK cells (Figure 1C), although mean IFN-γ expression per cell was similar (data not shown). Because NKG2A synergizes with specific inhibitory Ly49 receptors to educate mouse peripheral NK cells (Zhang et al., 2019), Ly49 might contribute to the superior functional competence of NKG2A^+^ uNK cells. To test this, we measured the percentage of cells expressing the self-specific educating Ly49I receptor in B6 mice (Fernandez et al., 2005). Because we found no significant difference in Ly49I expression between NKG2A^+^ and NKG2A^−^ subsets, we could rule out the potentially confounding effect of Ly49I education in NKG2A^+^ uNK cells (Figure 1C). DNAM-1 expression correlates with peripheral NK cell education (Wagner et al., 2017), and here we find that it was expressed in greater percentages of NKG2A^+^ compared with NKG2A^−^ uNK cells (Figure 1C). The greater functional competence of NKG2A^+^ uNK cells could be a secondary effect of advanced maturity of NKG2A^+^ uNK cells. KLRG1 and CD11b correlate with peripheral NK cell maturation. However, KLRG1 expression was found in similar percentages of NKG2A^+^ and NKG2A^−^ uNK cells (Figure 1C) and CD11b was higher for NKG2A^−^ NK cells (Figure 1C). This suggests that NKG2A^+^ uNK cells are not more mature than NKG2A^−^ uNK cells, as shown previously in human NK cells (Björkström et al., 2010); therefore, the superior functional response of NKG2A^+^ cells more likely results from education than from differences in maturation. This dataset shows that NKG2A-educated uNK cells are more functionally competent than NKG2A^−^ uNK cells in response to NK1.1 crosslinking.

### NKG2A is required for peripheral NK cell function

NK cells from NKG2A-deficient *Klrc1*^−/−^ mice display normal maturation, cell surface receptor repertoires, and cellular development (Rapaport et al., 2015), and we confirmed here that both uNK cells and CD8^+^ T cells developed in normal numbers in both uterus and spleen of *Klrc1*^−/−^ dams (Figures S1G and S1H). To confirm the contribution of NKG2A to peripheral NK cell education, we used a standard *in vivo* assay based on rejection of MHC-deficient *B2m*^−/−^ hematopoietic cells (Höglund and Brodin, 2010). Using this missing-self response assay (Figure 1D), we showed that *Klrc1*^−/−^ mice were almost 50% less efficient at rejecting MHC-deficient cells than B6 mice (Figure 1E), confirming the key role of NKG2A in peripheral NK cell education (Zhang et al., 2019). Altogether, these data indicate that NKG2A modulates NK cell education both systemically and in the uterus during pregnancy, demonstrating the suitability of NKG2A-deficient *Klrc1*^−/−^ mice to model NK cell education in pregnancy.

### NKG2A is required for uterine vascular adaptation to pregnancy

**Figure 2:**
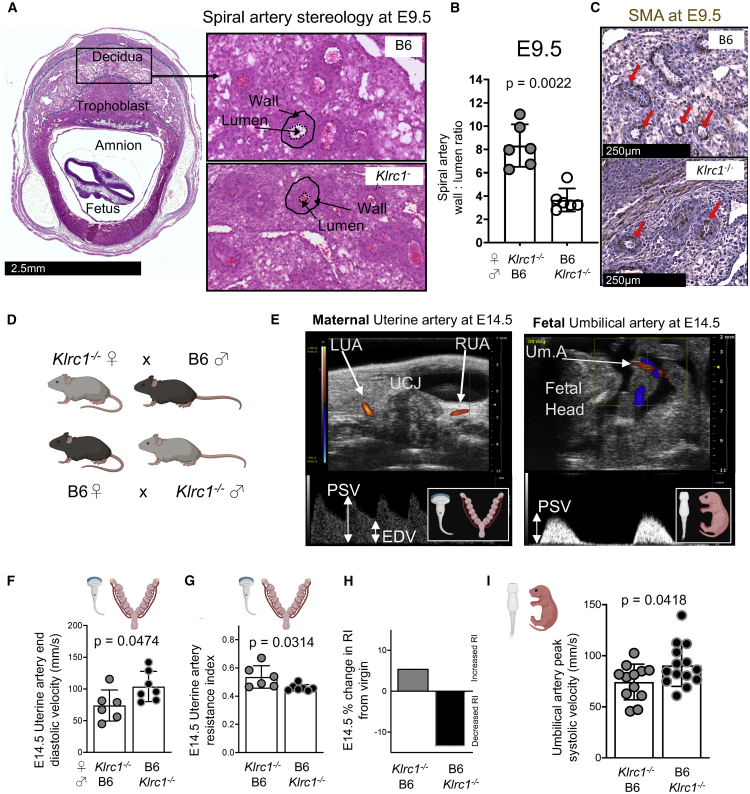
NKG2A is required for maternal and fetal vascular adaptation to pregnancy (A) Representative section of E9.5 implantation site of a B6 dam stained with H&E indicating, on the left, the decidua in relation to trophoblast, amnion, and fetus. Sections on the right show stereological assessment of wall and lumen (both indicated by black arrows) of spiral arteries from two representative dams of each genotype (1 experiment). (B) Quantification of the spiral artery ratio between vessel wall area and corresponding lumen area (each datapoint represents the mean of 15 measurements in one implantation site, n = 2–4 dams per group, t test). (C) Immunohistochemistry (IHC) staining for smooth muscle actin (SMA, brown) by strain, indicating relatively thicker-walled SMA-associated arteries of *Klrc1*^−/−^ dams (red arrows). (D) Mating strategy for ultrasound assessment of gestational hemodynamics in both dams (F–H) and fetuses (I) at E14.5. (E) Representative (2 experimental repeats) microultrasound color Doppler image of uterine artery (left) and umbilical artery (right). The left panel shows both uterine arteries (UAs) lateral to the utero-cervical junction (UCJ) above and, below, the pulsed wave (PW) Doppler waveform (PSV, peak systolic velocity; EDV, end diastolic velocity). The right panel shows a representative image of the uterine umbilical artery (red, Um.A, indicated by arrow) and the umbilical vein (blue), with the Um.A waveform below. (F) UA EDV and (G) UARI comparison at E14.5 by group (each data point represents one mouse, t test). (H) Percentage change in resistance index (RI) from virgin to E14.5 for each type of mating (bars show mean change in RI from data shown in (G) and Figure S2C. (I) Comparison of Um.A PSV at E14.5 by group (t test, each datapoint represents 1 fetus, n = 12–13 per group, t test). Scale bar in (A), 2.5 mm; scale bar in (C), 250 μm. Error bars in (B), (F), (G)m and (I) represent standard deviation.

The pattern of expression of both the receptor and its ligand in B6 dams (Figure S1) is ideally suited to test directly the hypothesis that maternal, not fetal, Qa-1 educates uNK cells through NKG2A and that NK cell education affects the outcome of pregnancy. In humans, maternal uterine spiral arteries undergo transformation in early pregnancy through the destruction of smooth muscle media by trophoblast cells to allow optimal fetal nourishment (Pijnenborg et al., 2006). In mice, transformation of the arteries relies primarily on IFN-γ produced by uNK cells (Ashkar et al., 2000). Because we observed reduced IFN-γ in B6 NKG2A^−^ uNK cells, we hypothesized that lack of uterine vascular adaptation would be suboptimal in the absence of NKG2A (Figure 2A). *Klrc1*^−/−^ dams displayed increased vascular wall area, normal lumen area, and more informatively, a two-fold greater size of the vascular wall relative to the lumen (Figure 2B; Figures S2A and S2B), with thicker smooth muscle actin (Figure 2C). This demonstrates that NKG2A is required for normal vascular remodeling, because the uterine arteries of *Klrc1*^−/−^ dams retain smooth muscle actin and thicker vessel walls relative to their lumen.

### NKG2A is required for maternal and fetal hemodynamic changes during pregnancy

We next assessed whether this vascular maladaptation leads to changes in upstream pressure indices in the maternal and fetal circulation. To do this, we compared vascular hemodynamics in dams and fetuses of (*Klrc1*^−/−^ ♀ x B6 ♂) and (B6 ♀ x *Klrc1*^−/−^ ♂) mating to directly compare genetically matched fetuses developing in either NKG2A-deficient or NKG2A-sufficient uteri. Because the fetuses were heterozygous in both sets of pregnancies, whereas the dams were either *Klrc1*^−/−^ or WT, this mating strategy allowed us to isolate the effect of the maternal genotype from that of the fetuses (Figure 2D). Using ultra-high-frequency microultrasound Doppler, we measured blood flow velocity in dams at E14.5 (left panel in Figure 2E)—by which time dilatation of the uterine artery because of spiral artery remodeling is expected (Rennie et al., 2016)—and in the umbilical artery in the same dams at E14.5 (right panel in Figure 2E). Although we observed no difference in baseline uterine artery resistance index (UARI) in a separate group of *Klrc1*^−/−^ and B6 virgin females (Figure S2C), end-diastolic blood flow velocity (UA EDV) was significantly reduced in pregnant *Klrc1*^−/−^ dams (Figure 2F). This contributed to significantly increased UARI (Figure 2G). Although both velocity and resistance indices appeared only mildly affected in *Klrc1*^−/−^ dams, the pregnancy-induced UARI reduction typically displayed by B6 dams did not occur in NKG2A-deficient dams (Figure 2H). Moreover, fetuses from *Klrc1*^−/−^ dams displayed abnormal umbilical blood flow (Figure 2I). This was characterized by a reduction in peak systolic velocity, because end-diastolic velocity is absent at this gestational age. These datasets show that NKG2A is required for normal hemodynamics in both maternal and fetal circulation and that these defects are determined by the maternal, not the fetal, genotype.

### NKG2A is required for optimal fetal growth

**Figure 3:**
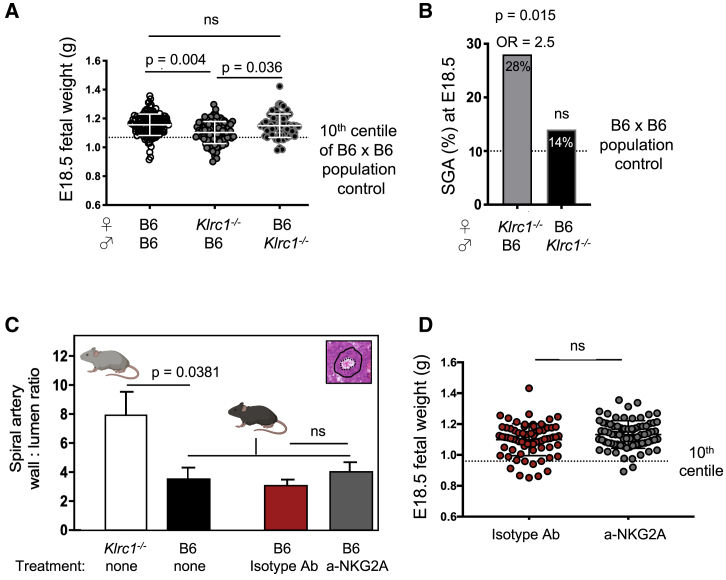
Maternal NKG2A is required for optimal fetal growth, but NKG2A blockade does not recapitulate the phenotype of *Klrc1*^−/−^ dams (A) Comparison of fetal weight at E18.5 by group (see also Table 1). The dotted line indicates the 10^th^ percentile of fetuses from the population control of fetuses from B6 x B6 pregnancies (mixed model analysis; each datapoint represents one fetus). (B) Frequency of small for gestational age (% SGA) E18.5 fetuses from *Klrc1*^−/−^ x B6 and B6 x *Klrc1*^−/−^ pregnancies compared with the baseline 10% SGA fetuses from B6 x B6 pregnancies (Fisher’s exact test; OR, odds ratio). (C) Visual representation of both sets of data presented in Figure 2B and Figure S3H to directly compare vascular changes, i.e., means and standard deviations of spiral artery wall:lumen ratios of untreated *Klrc1*^−/−^ dams (white bars), untreated B6 dams (black bars), and B6 dams treated with either isotype-matched control antibody (red bars) or blocking anti-NKG2A (gray bars). (D) Comparison of fetal weight at E18.5 in B6 dams treated with either isotype-matched control antibody or blocking anti-NKG2A, mixed model. The dotted line indicates the 10^th^ percentile of fetuses from B6 dams treated with isotype-matched control antibody (see also Table 2). Error bars in (A), (B), (C), and (D) represent standard deviation. See also Figure S3.

Low birthweight affects the health of human offspring even in the range of 5%–10% reduction (Juárez and Merlo, 2013), and dams with absent or hypofunctional uNK cells generate pups with weight reduction in the same range (Kieckbusch et al., 2014). To test whether the observed aberrations in both maternal and fetal hemodynamics caused by the absence of maternal NKG2A result in suboptimal fetal growth by the end of pregnancy (E18.5), we weighed 98 fetuses from 13 NKG2A-deficient *Klrc1*^−/−^ dams and 70 fetuses from 10 NKG2A-sufficient B6 dams. Both types of dams were mated with males of the reciprocal genotype. Fetuses from these two sets of dams were compared with a population control of 131 fetuses from 17 B6 dams mated with B6 males. Fetal weight was significantly reduced in NKG2A-deficient dams. The mean weight of fetuses from *Klrc1*^−/−^ dams displayed significant reduction compared with both the genotype control (1.10 ± 0.08 versus 1.15 ± 0.08, 4.3% reduction, p = 0.036) and the population control (1.16 ± 0.07, 5.2% reduction, p = 0.004) (Figure 3A; Table 1), suggesting that reduced fetal growth was driven by maternal NKG2A deficiency. In line with the results of other mouse strains with absent or hypofunctional NK cells, litter size was not affected in *Klrc1*^−/−^ dams (Figure S3A). The observed FGR in *Klrc1*^−/−^ dams was not affected by the size of their placentae, which showed normal weight (Figure S3B). Despite the reduction at term, fetal weight was normal in *Klrc1*^−/−^ dams at E15.5, consistent with the insufficient remodeling of the uterine arteries making the placenta of *Klrc1*^−/−^ unfit to meet the exponential fetal-growth demands typical of late pregnancy (Figure S3C). Fetuses developing in NKG2A-deficient dams were not only smaller overall but were also twice as likely to be classified as small for gestational age (SGA, defined as <10^th^ percentile of (B6 ♀ x B6 ♂) pregnancies) (Figure 3B). As many as 27/98 fetuses (28%) were SGA in NKG2A-deficient dams, whereas only 10/70 (14%) were SGA in the genotype control dams, which did not significantly differ from the 13/131 SGA fetuses (10%) of the population control (Figure 3B). The results show that maternal NKG2A is required for optimal fetal growth, and the extent of the growth reduction in dams lacking NKG2A is comparable to that of other strains of mice with absent or hypofunctional NK cells (Barber and Pollard, 2003; Ashkar et al., 2003; Kieckbusch et al., 2014; Boulenouar et al., 2016).

**Table 1:** Growth restriction in fetuses from *Klrc1*^−/−^ dams at E18.5

Group	Maternal strain	Paternal strain	Litters (n)	Fetuses (n)	Mean litter size	Mean fetal weight, g (SD)	∗p value
Population control	B6	B6	17	131	7.7	1.16 (0.07)	0.004
Study group	*Klrc1*^−/−^	B6	13	98	7.5	1.10 (0.08)	N/A
Fetal genotype control	B6	*Klrc1*^−/−^	10	70	7	1.15 (0.08)	0.036

∗ p values are in comparison to the study group composed of NKG2A-deficient Klrc1^−/−^ dams, mixed model. N/A, not applicable.

### NKG2A antibody blocking in WT dams does not phenocopy genetic NKG2A ablation

To distinguish between the effects of constitutive and those of acute absence of NKG2A signaling, we blocked NKG2A in B6 dams by administration of one dose of the blocking monoclonal antibody 20d5 (Vance et al., 1999; André et al., 2018) at E6.5 and assessed remodeling of spiral arteries at E9.5, as in Figures 2A and 2B. In another group of 19 B6 dams, we blocked NKG2A with two doses at E6.5 and E9.5 in 11 dams and assessed litter sizes and fetal and placental weights at E18.5, as in Figure 3A, comparing them with those of 8 isotype-control treated dams. We chose these two time points because the E6.5–E9.5 window is when uNK cells may exert their maximal function and when both uterine vasculature remodeling and placenta formation begin. Immune checkpoint blockade can result in fetal loss, as shown in dams treated with anti-PDL1, anti-CTLA-4, and anti-Tim-3 (Guleria et al., 2005; Xu et al., 2017). Although 2/11 dams treated with anti-NKG2A produced smaller litter sizes, the remaining 9 dams produced litters of normal size (Table 2; Figure S3D), indicating that the anti-NKG2A antibody did not obviously lead to embryonic lethality in this context. Fluorescence-activated cell sorting (FACS) analysis after the injections indicated the NKG2A epitope was 100% saturated with the administered antibody in both uNK and splenic NK cells up to three days after injection (Figure S3E; data not shown), suggesting NKG2A blockade for an appropriate duration, that is, at least up to E12.5, when remodeling of uterine arteries should be well under way. We then compared spiral artery remodeling in these NKG2A-blocked B6 dams with remodeling in NKG2A-deficient dams. Although vessel wall thickness was higher in the anti-NKG2A-treated group, the luminal area of spiral arteries was normal and the ratio between the areas of the wall and the lumen was not significantly different (Figures S3F–S3H). Thus, NKG2A blockade does not phenocopy the defective vascular remodeling of NKG2A-deficient dams (Figure 3C). Furthermore, the 71 fetuses from the 8 isotype-treated B6 dams and the 78 fetuses from the 11 anti-NKG2A-treated B6 dams displayed equivalent fetal weights at E18.5. We noted a non-significant 2.7% increase in mean fetal weight from the anti-NKG2A-treated dams (Figure 3D; Table 2). These results suggest that acute blocking of NKG2A does not phenocopy the effect of genetic NKG2A ablation on fetal weight and spiral arteries. In WT mice, before the blockade, NKG2A signaling had been functional and able to educate uNK cells in steady state, thus suggesting that NKG2A education of uNK cells before pregnancy may be sufficient to provide adequate uNK cell function during gestation, even when NKG2A signaling is acutely blocked during pregnancy.

**Table 2:** NKG2A-antibody blocking does not phenocopy NKG2A genetic ablation

Group	Maternal strain	Paternal strain	Injected at E6.5 and E9.5	Litters (n)	Fetuses (n)	Mean litter size	Mean fetal weight, g (SD)	p value
Isotype control	B6	B6	10 μg R35-95 (IgG2a)	8	71	8.9	1.10 (0.1)	N/A
Antibody treated	B6	B6	10 μg 20d5 (IgG2a)	11	78	7.1	1.13 (0.9)	ns

ns, not significant; N/A, not applicable.

### Asymmetric fetal growth and brain sparing in pups of NKG2A-deficient dams

**Figure 4:**
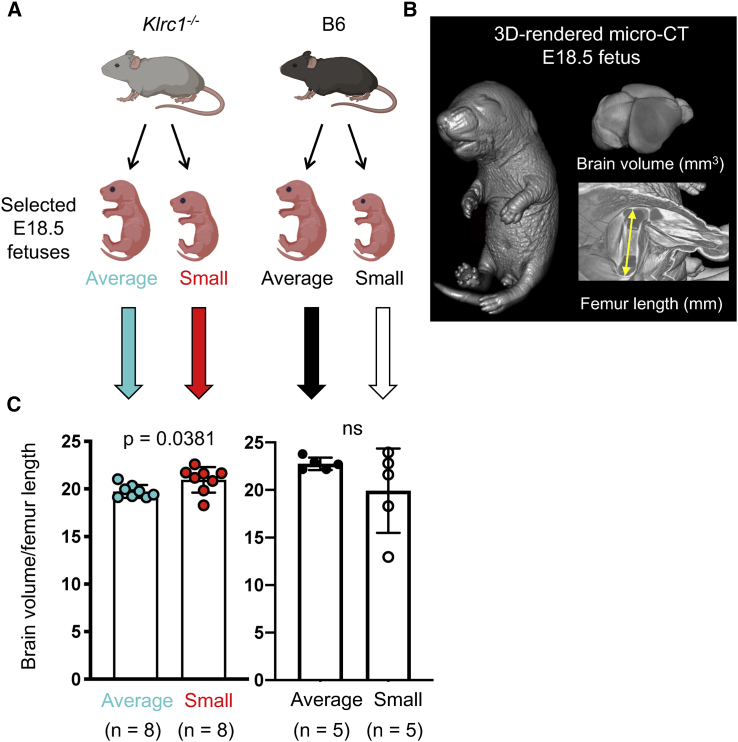
Asymmetric fetal growth and brain sparing in pups of NKG2A-deficient dams (A) Groups of selected average-weight and small E18.5 fetuses from litters of *Klrc1*^−/−^ dams (4 litters, 16 fetuses) and B6 dams (5 litters, 10 fetuses) used for micro-CT analysis. Mean fetal weights are shown in Figure S4A. (B) Representative micro-CT 3D-rendered images (from 2 experimental repeats) of an E18.5 fetus, its brain, and its femur. (C) Comparisons of brain volume: femur length ratios by group (each datapoint represents one fetus, t test). Error bars represent standard deviation. See also Figure S4.

Asymmetric fetal growth occurs in complicated pregnancies with inadequate maternal resources when the brain is preferentially spared from growth restriction at the expense of other fetal organs (Sharma et al., 2016). Fetal brain sparing is linked to cognitive and behavioral abnormalities in children (Tolsa et al., 2004; Eixarch et al., 2008; Oros et al., 2010) and is observed in mouse models (Cahill et al., 2014). Although there is variation in the growth of individual fetuses in normal litters, as shown in Figure 3A, SGA fetuses developing in normal pregnancies are expected to be healthy and grow symmetrically, similar to healthy human SGA babies. We hypothesized that the altered umbilical blood flow found at E14.5 in fetuses from *Klrc1*^−/−^ dams mated with B6 males (Figure 2I) was associated with subsequent asymmetric growth pattern in the smallest fetuses measured at the end of pregnancy (E18.5). To do this, we selected the smallest fetuses from each of 4 litters of *Klrc1*^−/−^ and 5 litters of B6 dams. These were compared with the average-weight fetuses within the litters of the same types of dams, as shown in Figure 4A. Fetal weights of the selected groups are indicated in Figure S4A. To accurately assess fetal brain development and symmetric body growth, we used micro-computed tomography (micro-CT) and compared ratios between brain volume and femur length (Figure 4B). Small fetuses from *Klrc1*^−/−^ dams had higher ratios than their average-weight littermates, whereas no difference was found between average-weight and small fetuses from B6 dams (Figure 4C). In other words, growth symmetry was found across all fetuses in B6 dams, in which both average-weight and small fetuses had brain volumes proportional to their femur length (Figure S4B). In contrast, the brains of small fetuses from *Klrc1*^−/−^ dams were disproportionally larger compared with their shorter femurs (Figure S4B). These results show that in a uterus with NKG2A-deficient uNK cells, the smallest fetuses display asymmetric growth and brain sparing and the origin of both was the maternal, not the fetal, genotype.

### NKG2A regulates the placental transcriptome

**Figure 5:**
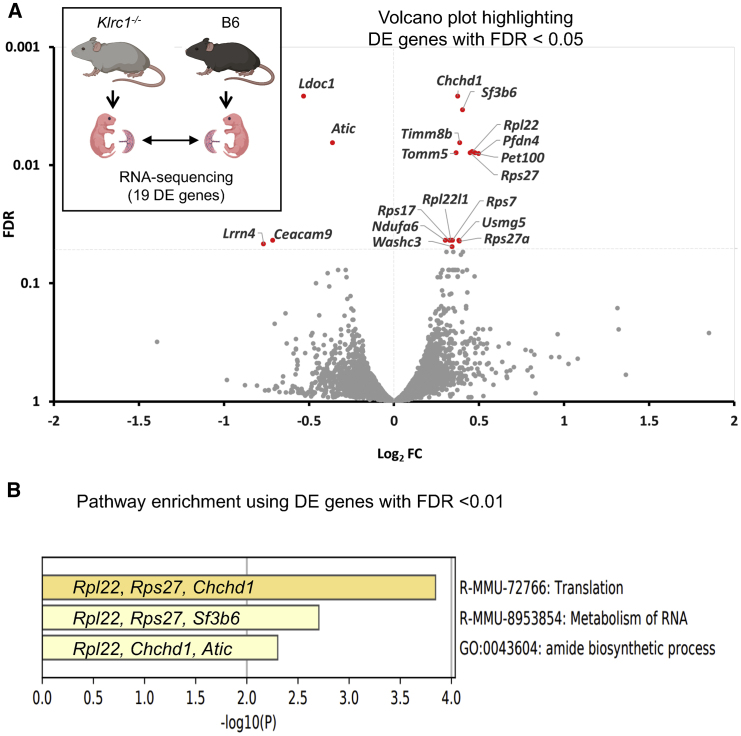
Maternal NKG2A regulates the placental transcriptome (A) Placentae of two E15.5 average-weight fetuses/litter in 5 litters of *Klrc1*^−/−^ dams and 5 litters of B6 dams, each mated with males of the reciprocal genotype, were selected for placental transcriptome analysis (n = 10 placentae/group, 6 males and 4 females in each group). The volcano plot shows the 19 differentially expressed (DE) genes in Log2FC (fold change) in the comparison between the placentae in *Klrc1*^−/−^ dams and those in B6 dams. A negative value indicates a lower relative expression in fetuses of *Klrc1*^−/−^ dams. FDR (false discovery rate) is the adjusted p value for multiple testing. See also Table S1. (B) Significantly enriched gene pathways identified by using DE genes with FDR < 0.01. The genes contributing to these pathways are indicated. Log10(p) is the p value in log base 10. See also Figure S5.

We aimed to determine whether the altered uterine and umbilical blood flow of NKG2A-deficient dams was associated with aberrant placental gene expression. We compared RNA expression in genetically matched placentae of (*Klrc1*^−/−^ ♀ x B6 ♂) and (B6 ♀ x *Klrc1*^−/−^ ♂) mating at E15.5 (n = 5 litters, 10 fetuses in each group). To isolate differences resulting from the presence or absence of maternal NKG2A, we matched fetal groups not only by gestational age, genotype, and sex composition but also by weight. Thus, we selected the placentae of the two fetuses closest to the mean fetal weight in each litter. The selection of average-weight fetuses was done to remove bias introduced by randomly selecting those who may be suffering from either FGR or fetal overgrowth. Previously reported mouse placenta housekeeping genes *Hprt*, *Gapdh*, *Actb*, *Ubc*, *Pol2ra*, and *Ywhaz* (Solano et al., 2016) were stably expressed by all placentae, across both groups, suggesting that these housekeeping genes did not bias the comparison (Figure S5). Supporting the robustness of our analysis, we found that all samples were only highly enriched in the gene expression signature titled placenta (data not shown). A list of all processed, filtered gene expression scores can be found in Data S1. The volcano plot in Figure 5A highlights the 19 differentially expressed (DE) genes reaching statistical significance with a false discovery rate (FDR) < 0.05, of which 14 were overexpressed and 5 were downregulated in placentae of *Klrc1*^−/−^ dams. Table S1 lists these 19 DE genes and highlights the top 10 DE genes with FDR < 0.01. Eight of these 10 genes were overexpressed in the placentae of *Klrc1*^−/−^ dams: ribosomal proteins *Chchd1*, *Rpl22*, and *Rps27*; the *Sf3b* unit of the ribonucleotide complex of spliceosomes; the *Pfdn4* chaperone that helps correct folding of nascent polypeptides; mitochondrial protein transporters *Timm8b* and *Tomm5*; and the chaperone of mitochondrial cytochrome *c* oxidase *Pet100*. The two DE genes with FDR < 0.01 downregulated in placentae of *Klrc1*^−/−^ are the tumor suppressor gene and regulator of nuclear factor κB (NF-κB) signaling *Ldoc1* and the transferase involved in purine biosynthesis *Atic*. Using these top 10 DE genes with FDR < 0.01, we performed a gene functional annotation analysis in Metascape to discover enriched pathways (Zhou et al., 2019; Figure 5B). The gene cluster *R-MMU-72766 Translation* was the most enriched in the analysis (Log10(p) = −3.86) and included *Rpl22*, *Rps27*, and *Chchd1*. *Rpl22*, *Rps27*, and *Sf3b6* contribute to *mRNA processing* (Log10(p) = −2.96) and *Metabolism of RNA* (Log10(p) = −2.71), whereas *Rpl22*, *Chchd1*, and *Atic* contribute to *Amide biosynthetic process* (Log10(p) = −2.28) (Figure 5B). These results suggest that RNA biology, protein synthesis, and translation are affected in placentae of dams whose uterus had NKG2A-deficient uNK cells.

### Lower risk of pre-eclampsia in women genetically programmed to NK cell education via NKG2A

Our results in mice suggest that NKG2A drives uNK cell education and that lack of maternal NKG2A in dams, but not in fetuses, affects utero-placental arterial remodeling, hemodynamics, and placental gene expression with downstream effects on fetal growth and brain development. These are features of pre-eclampsia, a heterogeneous, polygenic, and multifactorial syndrome that affects up to 1/10 pregnancies in certain human populations. We therefore set out to investigate whether women carrying the −21T allotypes of *HLA-B* that do not favor NKG2A education in humans are at greater risk of developing pre-eclampsia. To do this, we assessed the association with pre-eclampsia of the SNP rs1050458 on chromosome 6 (6p21), coding the −21M or −21T *HLA-B* dimorphism in a genome-wide meta-analysis of 9,515 maternal pre-eclampsia cases and 157,719 controls from multiple cohorts (Steinthorsdottir et al., 2020). The analysis revealed that the *G* allele coding the −21T *HLA-B* variant was associated with increased pre-eclampsia risk (p = 0.02). Although this association was not apparent in a smaller cohort of Central Asian mothers (p = 0.44), it was present in 7,219 European cases and 155,660 controls (p = 0.005, odds ratio [OR] = 1.07, 95% confidence interval [CI] 1.02–1.12) (Table 3). The data show that the *G* allele conferred a 7% risk in Europeans (95% confidence interval, 2%–12%). This result suggests that women genetically programmed to educate NK cells through HLA-B leader peptides that allow HLA-E to engage NKG2A are at lower risk of pre-eclampsia.

**Table 3:** Binomial logistic regression model, with the *G* allele predicting the outcome of human pregnancies

Cohort	*G* allele in population (%)	Beta = log(OR)	SE (beta)	OR	95% CI	p value
All	68	0.050	0.022	1.05	1.01–1.10	0.025
Central Asian	80	−0.042	0.055	0.96	0.86–1.07	0.444
Northern European	65	0.068	0.024	1.07	1.02–1.12	0.005

Beta, regression coefficient; OR, odds ratio; SE, standard error; CI, confidence interval. Sample sizes: All = 9,515 cases and 157,719 controls (dataset EGAD00010001988), of which the Central Asian cohort = 2,296 cases and 2,059 controls (dataset EGAD00010001984) and the Northern European cohort = 7,219 cases and 155,660 controls (dataset EGAD00010001984).

## Discussion

Although several inhibitory receptors can educate NK cells, we show here that NKG2A is required for optimal uNK cell education in mice and its absence has repercussions on utero-placental vascular dynamics, fetal growth, and brain development. Some of these features underpin the human syndrome pre-eclampsia. Our analysis of a cohort of >7,000 European pre-eclampsia cases revealed that in certain human populations, the −21 HLA-B→HLA-E→NKG2A pathway may contribute to pre-eclampsia risk. This evidence establishes the importance of NK cell education in physiology and suggests that weak NKG2A education is linked to disease risk.

Our results in mice showed that maternal education of uNK cells achieved through NKG2A was required for optimal uNK cell function. In both humans and mice, NKG2A is expressed in a heterodimer complex, together with CD94. A redundancy for NKG2A education is shown in a CD94-deficient model in 129/SvJ mice, in which NKG2A is not expressed (Orr et al., 2010). However, 129/SvJ mice have more inhibitory Ly49 receptors that can educate NK cells than B6 mice, thus compensating for the lack of NKG2A-driven education (Orr et al., 2010). Importantly, synergy between NKG2A and Ly49 receptors occurs to educate NK cells to recognize and reject missing self in B6 mice (Zhang et al., 2019). We showed that NKG2A^+^ uNK cells expressed more DNAM-1 and produced more IFN-γ than NKG2A^−^ uNK cells upon NK1.1 crosslinking, suggesting that they were better educated. IFN-γ is a key factor for arterial remodeling in mouse pregnancy (Ashkar et al., 2000). Although human uNK cells produce cytokines and chemokines, there is little IFN-γ production unless they are stimulated *in vitro* (King et al., 1989; Hanna et al., 2006). Despite anatomical differences between human and murine utero-placental tissues, our observed association of reduced spiral artery remodeling with increased uterine artery resistance in *Klrc1*^−/−^ dams is in keeping with established human studies of defective placentation, in which FGR and disorders of gestational hypertension such as pre-eclampsia are common (Olofsson et al., 1993; Sağol et al., 1999). All human fetuses lying below the 10^th^ percentile of estimated fetal weight are classified as SGA. Within these, some are constitutionally small and healthy, whereas others display features of pathological FGR. Defective placentation is the likely underlying mechanism in most cases of FGR and pre-eclampsia. Human fetuses suffering from pathological FGR *in utero* can display characteristic responses to an abnormal placental supply of nutrients and oxygen through changes in umbilical artery pressure indices and a relative increase in fetal middle cerebral artery blood flow (Robson et al., 2013). This relative increase in blood flow to the brain is known as fetal cerebral redistribution or brain sparing and is thought to contribute to asymmetric FGR (e.g., increased head:abdominal circumference) (Sharma et al., 2016). Although it might be seen as an advantageous compensation, brain sparing in fetal development could lead to cognitive and behavioral abnormalities in children affected by asymmetric growth restriction (Tolsa et al., 2004; Eixarch et al., 2008; Oros et al., 2010). Our findings of asymmetric FGR in fetuses of *Klrc1*^−/−^ dams make them a suitable model to test the effect of brain sparing in the offspring.

We also found variations in the transcriptome between placentae of *Klrc1*^−/−^ dams and that of B6 dams, even though their heterozygous fetuses are genetically identical. Enrichment of gene pathways associated with protein translation fitted the model that abnormal remodeling of decidual blood vessels and placental perfusion leads to placental oxidative stress, a key pathophysiological determinant of pre-eclampsia (Hung et al., 2002). Protein translation, essential for normal development, is inhibited in placental tissue from human pregnancies affected by states of hypoxia at altitude (Yung et al., 2012) and in those placentae affected by pre-eclampsia and growth restriction (Yung et al., 2008). This suppression of protein synthesis is thought to be mediated by cell stress, secondary to altered perfusion of the placenta (Burton and Jauniaux, 2018). In our placental transcriptomics analysis, the gene showing the greatest increase in placental expression from NKG2A-deficient dams was ribosomal protein *Chchd1*, which is implicated in embryonic and placental growth and in brain development in *Chchd1*^−/−^ mice (Mouse Genome Informatics and the International Mouse Phenotyping Consortium, 2014). Mitochondrial ribosomal proteins *Timm8b* and *Tomm5* and the chaperone of mitochondrial cytochrome *c* oxidase *Pet100* were also upregulated, with the latter previously linked to FGR in a patient who displayed a truncated version of the gene (Oláhová et al., 2015). These genes are significantly upregulated in dams lacking NKG2A, in keeping with cellular stressors inducing overexpression of ribosomal proteins. This suggests that weak uNK cell education may expose the maternal-fetal interface to stress, probably linked to perturbed utero-placental hemodynamics. The gene showing the greatest decrease in placental expression from NKG2A-deficient dams was the tumor suppressor gene and regulator of NF-κB signaling *Ldoc1*, which plays a role in normal placental cell differentiation and maturation (Nagasaki et al., 2003; Duzkale et al., 2011).

We also show that fetuses from *Klrc1*^−/−^ dams had a lower mean body weight at the end of gestation and are more likely to fall below the 10^th^ percentile for their gestational age. This finding was independent of placental weight and supports the evidence that NKG2A contributes to the optimal delivery of nutrients and oxygen to the fetal circulation. Blocking the NKG2A receptor with a monoclonal antibody in early pregnancy had an effect on the arterial wall area, which was significantly greater than that on the arterial wall area in isotype-treated WT dams. These results combined suggest that the remodeling of the spiral arteries was suboptimal in NKG2A-treated dams; however, there was no significant effect on the arterial wall area relative to luminal wall area. This suggests that blood flow after antibody treatment is not affected to the same extent as in NKG2A-deficient mice. In line with this, no significant effect of the antibody treatment was seen on fetal growth. Thus, NKG2A genetic ablation, but not NKG2A-antibody blockade, impairs spiral artery adaptation to pregnancy and fetal weight. In other words, acute blockade of NKG2A during early gestation does not seem to negate the contribution of pre-existing and constitutive NKG2A signaling that mediates uNK cell education. This suggests that loss of uNK cell inhibition may not negatively affect pregnancy outcome, unlike constitutive loss of education in NKG2A-deficient dams. Peripheral NK cells lose inhibition upon acute NKG2A blockade, unleash their potential, and kill Qa-1-expressing cancer cells (André et al., 2018). Mouse trophoblast does not appear to express the NKG2A ligand Qa-1; hence, the only source for either education or inhibition through NKG2A is maternal cells.

Although the primary causes of pre-eclampsia are unclear, susceptibility to the disease has a genetic basis. Heritability of maternal pre-eclampsia is estimated to be 38.1% in Europeans and 54% in Central Asians (Steinthorsdottir et al., 2020). However, little is certain about the identity of maternal or fetal genes causing pre-eclampsia. Candidate gene approaches and GWAS have identified several candidate genes that might influence pre-eclampsia risk, but many studies lack statistical power or could not be replicated in independent populations (Johnson et al., 2012; Zhao et al., 2012). A recent genome-wide meta-analysis of Northern European and Central Asian mothers and offspring from pre-eclamptic pregnancies has identified 5 maternal genetic variants near genes involved in blood pressure regulation (Steinthorsdottir et al., 2020) and one fetal susceptibility locus near *FLT1* (McGinnis et al., 2017). Using the maternal dataset in this meta-analysis, we found association of pre-eclampsia with the maternal *G* allele of the rs1050458 SNP on chromosome 6 coding the −21T variant of the *HLA-B* gene. The rs1050458 SNP was not in linkage disequilibrium with any of the 5 recently discovered pre-eclampsia-associated sequence variants in maternal genomes on chromosomes 3, 4, 12, 16, and 20 (Steinthorsdottir et al., 2020). The 7% risk conferred by −21T HLA-B has an effect size comparable with that of genes involved in the control of blood pressure in Europeans (9%–12%, Steinthorsdottir et al., 2020) or genetic determinants of other reproductive traits (Day et al., 2016). Because the −21M/T HLA-B dimorphism determines high and low HLA-E expression, respectively, this in turn determines that NKG2A is the NK receptor favoring education in −21MT and −21MM individuals (Horowitz et al., 2016). This genetic evidence, together with the results of our mouse studies and the GWAS analysis, implicates NKG2A and its pathway in pre-eclampsia. It remains to be tested in future studies to what extent this dimorphism affects human uNK cell function. Because trophoblast does not express HLA-B, fetal HLA-E expression is driven by HLA-C and/or pregnancy-specific HLA-G. This may also modulate the function of NKG2A-educated uNK cells, for example, by engaging with the activating NKG2C receptor. The frequency of the −21M *HLA-B* allele is highest in Europe; intermediate in Africa, Asia, America, and Polynesia; and lowest in Australia (Horowitz et al., 2016). Ultimately, further replication in both European and other ancestry groups will be important, particularly in view of disparate linkage disequilibrium of −21M *HLA-B* alleles with *HLA-C* alleles in various populations. It will be interesting to study the relative contribution of KIR-driven and NKG2A-driven education on uNK cell function and the role of specific KIR. For example, NKG2A synergizes with KIR3DL1, but not with KIR2DL1 or KIR2DL3, in peripheral NK cell education (Sim et al., 2016). It will also be important to integrate the findings reported here on NKG2A in pre-eclampsia with the established association of certain maternal KIR and fetal HLA-C variants with pre-eclampsia (Moffett and Colucci, 2015).

We conclude that NKG2A is a key regulator of uNK cell function and contributes to reproductive fitness. We have provided insights into its role in human and murine pregnancy. By using clinically translatable pregnancy outcome measures, we have provided further evidence of how immune models of FGR can recapitulate the pathophysiological processes, such as asymmetric FGR and brain sparing, displayed in human pregnancies. More broadly, we have provided evidence that NK cell education governs physiological processes.

### Limitations of study

Although we thoroughly investigated the impact of NKG2A genetic ablation in dams and their fetuses, we did not directly compare uNK cell function *in vitro* from B6 and *Klrc1*^−/−^ mice. This approach is ongoing. The results of placental gene expression should be validated to further investigate the observed gene pathway dysregulation. We also did not assess the role of the HLA-B −21 dimorphism on human uNK cell function *in vitro*. This work is ongoing and could lead to a greater understanding of the mechanisms influencing our observations in the reported GWASs. Finally, the genetic linkage of −21M dimorphism with pre-eclampsia was found in European populations, not the Asian cohort. The reason for this is currently unclear, and the linkage should be tested in other populations. Nevertheless, preliminary work that suggests an interaction between NKG2A and KIR genes may influence disease risk.

## STAR★Methods

### Key resources table

**Table:** 

REAGENT or RESOURCE	SOURCE	IDENTIFIER
**Antibodies**		

CD49a(Ha31/8)	BD Biosciences	Cat 562115; RRID: AB_11153117
NK1.1(PK136)	BD Biosciences	Cat 562864; RRID: AB_2737850
NKp46(29A1.4)	Thermofisher	Cat 46-3351-82; RRID: AB_1834441
CD3 (17A2)	Biolegend	Cat 100232; RRID: AB_2562554
CD11b(M1/70)	Biolegend	Cat 101239; RRID: AB_11125575
CD19(1D3)	BD Biosciences	Cat 564296; RRID: AB_2716855
CD45(30-F11)	BD Biosciences	Cat 564279; RRID: AB_2651134
NKG2A B6(16A11)	Biolegend	Cat 142807; RRID: AB_11125166
NKG2A/C/E (20d5)	BD Biosciences	Cat 550520; RRID: AB_393723
Qa-1b(6A8.6F10.1A6)	Miltenyi Biotec	Cat 130-104-219; RRID: AB_2653013
EOMES(Dan11mag)	Thermofisher	Cat 48-4875-82; RRID: AB_2574062
CD8a(53-6.7)	Biolegend	Cat 100743; RRID: AB_2561352
Ly49-I(YLI-90)	Thermofisher	Cat 12-5895; RRID: AB_466022
IFN-gamma(XMG1.2)	Thermofisher	Cat 17-7311-82; RRID: AB_469504
Smooth Muscle Actin	Agilent	Cat M0851; RRID: AB_2223500
TruStain FcX (anti-mouse CD16/32) Antibody(93)	Biolegend	Cat 101319; RRID: AB_1574973
Ultra-LEAF Purified anti-mouse NK-1.1(PK136)	Biolegend	Cat 108757; RRID: AB_2800567
Purified Rat anti-mouse NKG2A/C/E(20d5)	BD Biosciences	Cat 550518; RRID: AB_393721
Purified Rat IgG2a, κ Isotype Control(R35-95)	BD Biosciences	Cat 553927; RRID: AB_395142

**Chemicals/reagents**		

CellTrace CFSE Cell Proliferation Kit, for flow cytometry	Thermofisher	Cat C34554
Phosphate Buffered Saline 1X	Thermofisher	Cat 10010023
Phosphate Buffered Saline 10X	Thermofisher	Cat 70011044
Dubecco’s PBS 1X (no calcium/magnesium)	Thermofisher	Cat 14190144
Hank’s Balanced Salt Solution 1X	Thermofisher	Cat 15266355
0.5M EDTA	Thermofisher	Cat 15575020
RPMI 1640	Thermofisher	Cat 11875093
Fetal bovine serum (heat inactivated)	Thermofisher	Cat 10082147
Protein transport inhibitor cocktail 500x	Thermofisher	Cat 00-4980-93
Penicillin-streptomycin	Sigma-Aldrich	Cat P4333
Liberase DH	Roche	Cat 5401054001
Percoll solution	Fisher Scientific	Cat 10166144
RBC lysis buffer 10X	Biolegend	Cat 420302
BD Brilliant Stain Buffer	Fisher Scientific	Cat 15349374
Sodium Azide	Sigma-Aldrich	Cat 71290
Bovine serum albumin	Sigma-Aldrich	Cat A0281
Ultracomp ebeads	Thermofisher	Cat 01-2222-42
Permeabilisation buffer 10X	Thermofisher	Cat 00-8333-56
Fix/perm buffer set	Thermofisher	Cat 88-8824-00
16% w/v PFA, methanol-free	Fisher scientific	Cat 11490570
40% acrylamide solution	Bio-Rad	Cat 161-0140
2% bis-acrylamide solution	Bio-Rad	Cat 1610142
VA-044 initiator	WAKO chemicals	Cat 925-41020
Saponin	Sigma-Aldrich	Cat 47036
RNeasy Plus Universal Mini Kit	QIAGEN	Cat 73404
RNAlater	Thermofisher	Cat AM7021
Zymo RNA Lysis buffer - (50ml)	Zymo Research	Cat R1060-1-50
Omnipur sterile water	Sigma-Aldrich	Cat 9602
Agarose	Sigma-Aldrich	Cat A9539
Iodine 0.05M	Sigma-Aldrich	Cat 70231

**Experimental models: organisms/strains**		

Mouse: *Klrc1*^*−/−*^	Rapaport et al., 2015	n/a
Mouse: C57BL/6	Charles River	n/a

**Software and algorithms**		

GraphPad Prism 8 software	GraphPad	https://www.graphpad.com/
Amira 6.3 software	FEI	https://www.fei.com/home/
FlowJo v.9.3.2	TreeStar	https://www.flowjo.com
IBM SPSSv21 software	IBM	https://www.ibm.com/us-en/?ar=1

### Resource availability

#### Lead contact

Further information and requests for resources and reagents should be directed to and will be fulfilled by the Lead Contact, Francesco Colucci (fc287@medschl.cam.ac.uk).

#### Materials availability

This study did not generate any new materials, reagents or mouse strains.

#### Data and code availability

Processed gene expression data from RNA-sequencing comparisons (Figure 5) are available in Data S1, and raw unprocessed files are freely available at Gene Expression Omnibus (https://www.ncbi.nlm.nih.gov/geo/). Data used for analysis of human cohorts is publicly available at https://ega-archive.org/.

### Experimental model and subject details

#### Animals

Mice used for flow-cytometry were bred at the University of Cambridge Central Biomedical Service (CBS, pathogen-free), others were either bred at CBS or the University of Cambridge Combined Animal Facility (CAF). All mice were housed according to UK Home Office guidelines. Mouse experiments were approved by the University of Cambridge Ethical Review Panel and carried out in accordance with Home Office Project License PPL 70/8222. C57BL6 (B6) mice were purchased from Charles River, UK (CBS) or Envigo, UK (CAF). Mice with a targeted *Klrc1* deletion generated on a C57BL/6 background (*Klrc1*^−/−^) (Rapaport et al., 2015) lacking NKG2A were a kind gift from the Helmholtz-Zentrum München institute. Only female mice were used for the *in vivo* rejection assay outlined in Figure 1D. For all flow cytometry, spiral artery and fetal weight comparisons animals were housed in IVC cages in pathogen-free conditions under standard husbandry. Otherwise, animals were housed in standard cages under standard husbandry conditions. More information regarding the *Klrc1*^−/−^ strain used throughout the paper can be found here: http://www.informatics.jax.org/allele/key/877287.

#### Human studies

We used a recently published meta-analysis of eight GWAS of European and Central Asian cohorts to determine whether SNP rs1050458 encoding the −21T variant of the leader peptide of HLA-B is associated with increased risk of pre-eclampsia (Steinthorsdottir et al., 2020). All pregnancies were singleton. Pre-eclampsia was clinically defined as recent-onset hypertension > 20th week of gestation, with blood pressure ≥ 140 mmHg (systolic) or ≥ 90 mmHg (diastolic) on at least two occasions; and recent-onset proteinuria of 0.3 g/24 h or more, or ≥ 1+ on dipstick analysis of urine. Pregnant women with history of essential hypertension, diabetes or chronic renal disease were excluded from the study. Meta-analyzed as well as individual-level GWAS data were generated by the InterPregGen Consortium (Morgan et al., 2014; PMID: 26568652) and are deposited in the European Genome-phenome Archive (https://ega-archive.org).

### Method details

#### Flow cytometry and education assays

i) Phenotyping: Whole uteri and spleens were processed according to previously described protocols (Collins et al., 2009) using Liberase DH (Roche) to conserve the NKG2A epitope during enzymatic digestion.
ii) *In vitro* activation assays: Uterine tissue was processed as described above for phenotyping, however leukocytes were enriched on an 80%/40% Percoll (GE Healthcare Life Sciences) gradient prior to stimulation. For stimulation, cells were added to wells pre-coated with anti-NK1.1 antibody (PK136, Biolegend) (20 μg/ml) for 9.5 hours, in 500 μL complete media. Cells were incubated with either anti-NK1.1 antibody + protein transport inhibitor (PTI) (added after 1 hour) or PTI only, at 37°C.
iii) Cell labeling: Conjugated antibodies were diluted in *Horizon Brilliant Stain Buffer* (BD Biosciences) for extracellular antigens, or 1x permeabilisation buffer (eBioscience) for intracellular staining. Approximately 1x10^6^ live cells were incubated with 25μl *TruStain* (Biologend) anti-CD16/32 before being stained with the following antibodies(clone): CD49a(Ha31/8), NK1.1(PK136), NKp46(29A1.4), CD3 (17A2), CD11b(M1/70), CD19(1D3), CD45(30-F11), NKG2A B6(16A11), NKG2A/C/E (20d5), Qa-1b(6A8.6F10.1A6), EOMES(Dan11mag), CD8a(53-6.7), Ly49-I(YLI-90), IFN-gamma(XMG1.2) DNAM-1 (TX42.1) and KLRG1 (2F1). Cell viability was ascertained by labeling with fixable viability dyes (eBioscience). For most experiments, we used the 20d5 antibody to detect NKG2A as all NK cells that stain with 20d5 also stain with 16A11, and like others, we found no evidence that NKG2-C and -E are expressed on normal B6 NK cells (Vance et al., 1999; Rapaport et al., 2015).
iv) Acquisition and analysis: Samples were acquired on an LSRFortessa (BD) and analyzed using FlowJo (Treestar) software. Values for IFN-γ in paired uterine activation analyses were expressed as (% in anti-NK1.1 - % in untreated).
v) *In vivo* rejection assays: Whole spleens were collected from donor *B2m*^*−/−*^ and B6 females, mechanically processed and stained with Carboxyfluorescein Diacetate Succinimidyl Ester (CFSE) for 20 minutes at 37°C. A 50:50 suspension of *B2m*^*−/−*^ (5mM CFSE) and B6 (0.5mM) splenocytes were injected into recipient mice and spleen harvested 48 hours later. The ratio of CFSE +:- cells ascertained through flow cytometry.

#### Spiral artery remodelling

In E9.5 females, whole implantation sites were collected and formalin-fixed prior to quantitative H&E stereological analysis, as previously described (Kieckbusch et al., 2015). In the same implantation sites, immune-histochemical analysis of smooth muscle actin expression was performed as previously described (Boulenouar et al., 2016). Sections were scanned using the *Nanozoomer* digital slide scanner (Hamamatsu) and analyzed using *NDP.view2* (Hamamatsu) software. Spiral artery wall/lumen area was measured with the assessor blinded to maternal genotype.

#### High frequency micro-ultrasound measurements

Mice were anaesthetised using 5% isofluorane and placed on a heat mat, as previously described (Zhang and Croy, 2009; Greco et al., 2013). A *Vevo 2100* micro-ultrasound machine was used with a MS500D transducer probe. Implantation sites were identified and orientated in B-Mode, whereas color Doppler was used to identify blood vessels. Blood flow velocity data was retrieved in PW Doppler mode (40MHz frequency, 10 kHz PRF, Doppler angle of insonation < 40 degrees). The ultrasound operator was blinded to the mouse strain.

Single uterine arteries were identified in virgin mice to minimize anesthetic exposure, whereas both right and left were measured (and averaged) in pregnant mice to control for variable numbers of concepti in each uterine horn. Peak systolic velocity (PSV) and End Diastolic Velocity (EDV) were recorded over 3 cardiac cycles, and averaged. Resistance index was calculated as (PSV/(PSV-EDV) (Arbeille et al., 1983). Umbilical arteries were identified in one random fetus per uterine horn, per dam. No umbilical EDV was present at E14.5, which has also been reported in CD-1 mouse fetuses (Hernandez-Andrade et al., 2014), so pulsatility was not calculated.

#### Micro-CT measurement of fetuses

Fetuses were euthanized and weighed at E18.5. After all fetuses in each litter (n = 4-5 litters) were weighed, the fetuses closest to the litter mean (1-2 per litter) and the lightest fetuses (1-2 per litter) were selected for analysis. Fetuses were then processed through previously established protocols (Wong et al., 2012, 2013). Briefly, fetuses were fixed in 4% paraformaldehyde before undergoing hydrogel hybridization and iodine staining. Fetuses were scanned in 1% agarose in a *Nikon XT 225 micro-CT* at the Department of Zoology, University of Cambridge (voxel length was fixed at 14.7 microns) within 1 week of collection. Image stacks were processed in Amira 6.3 (*FEI*) software. Brain volume was manually segmented through voxel intensity threshold selection to visualize organ boundaries. Brain volume segmentation included olfactory lobes, cortices, cerebellar and medullae (segmented at superior aspect of spinal cord). Femur length was measured along the diaphysis, from the distal tip of the medial condyle to the proximal tip of the head of femur.

#### RNA-sequencing

We aimed to characterize any differences resulting from the presence or absence of maternal NKG2A through matching fetal comparison groups by gestational age, weight, sex composition and genotype. Sample size was based on previous studies (Chu et al., 2016, 2019). All E15.5 placentae were collected in *RNALater* (Thermofisher) and stored at 4°C for 3-4 days, after corresponding fetal weight was recorded. One quarter (placental disc halved, and halved again) of each placenta was used for RNA extraction in the following process: tissue was homogenized in *RNA lysis buffer* (Zymo Research, CA, USA) for 2 × 20 s using an *MP FastPrep-24 Tissue and Cell Homogenizer* (Hyland Scientific, WA, USA). RNA was purified using *RNAeasy Plus Universal Mini Kit* (QIAGEN, MD, USA) columns and protocols. All placentae were processed fresh at the same time. Both groups contained an equal split of extracted RNA sequenced immediately, and after 1 year in storage. We ran one placental sample twice - once fresh and once after being frozen, and detected a small batch effect, which was corrected for (see below). The sex of each fetus was determined through the identification of male-specific chromosomes. Each group contained 40% female and 60% male fetuses, so no batch correction for fetal sex was performed. All total RNA samples underwent initial quality control using a standard Bioanalyzer protocol (Agilent, CA, USA). Gel electrophoresis to check RNA quality and integrity was performed using RNA ScreenTape Analysis (Agilent), with all samples achieving a RNA Integrity score (RINe) > 8. The coding transcriptome was captured and cDNA libraries prepared using TrueSeq Stranded mRNA (Ilumina), and pooled samples were sequenced on a NextSeq 500 system (Ilumina). The loading concentration was 1.8pM, and 1% Phix (Ilumina) was run as a control. The kit used for sequencing was a High Output 75 cycle kit (Ilumina). The fastq samples were run through quality control checks, trimmed to remove low quality bases and adapters and mapped to the mouse genome (Dobin et al., 2013). Mapped reads were counted to determine the amount of expression in each gene using HTSeqCount (Anders et al., 2015), counted reads were loaded into the edgeR package in R software (Robinson et al., 2010), counts were filtered (using 5CPM), GC corrected using the CQN package (Hansen et al., 2012) and normalized on a per comparison basis, then differential expression was determined using an edgeR generalized linear model, which took into account the batch effect of the samples being compared, by using SVA (Leek et al., 2012). The p values were corrected for multiple testing and finally sample clustering relationships (PCA) were analyzed using the R package DESeq2 (Love et al., 2014). Normalized expression values (CPM) were used as input data for SaVanT (Lopez et al., 2017) to produce enrichment scores on mouse gene expression signatures (http://biogps.org/), as previously described (Chu et al., 2019). The 10 genes with an FDR < 0.01, were considered to be DE and were entered for functional pathway enrichment using a gene annotation and analysis resource (http://metascape.org). Pathways were considered to be enriched if they displayed a p value < 0.01, a minimum gene count of 3, and an enrichment factor > 1.5 (the enrichment factor is the ratio between the observed counts and the counts expected by chance) were collected and grouped into clusters based on their membership similarities.

#### NKG2A blockade

Mice were injected at E6.5 and E9.5 for fetal weight experiments, and E6.5 for spiral artery remodelling assays. Pregnant mice at E6.5 were first placed inside a warming box to encourage peripheral vasodilation. They were then placed inside a restrainer to undergo tail vein injection. These mice were identified through ear-notching, and injected again 3 days later at E9.5 for fetal weight analysis. Mice were injected with either 10μg 20d5 (IgG2a) anti-NKG2A/C/E or 10μg R35-95 (IgG2a) isotype control diluted in 50μl sterile 1xPBS, by intravenous tail vein injection.

### Quantification and statistical analysis

In general, Shapiro-Wilk test for normality (alpha = 0.05) was applied to all datasets except RNA-seq and fetal and placental weight. For comparison of means across two groups a parametric test was used if both groups passed normality testing. If either or both groups failed normality testing, a Mann-Whitney test was used. For multiple group comparisons, a one-way ANOVA was used for normally distributed data with post hoc t tests corrected for multiple comparisons. All p values are two-sided, unless stated. If a p value was < 0.05, ‘ns’ was stated as not significantly different.

#### RNA-sequencing –

We used the exact test edgeR approach to make pairwise comparisons between groups. We then adjusted for multiple testing via the FDR (Benjamini-Hochberg) approach used by edgeR (Robinson et al., 2010).

#### Mouse fetal and placental weight –

Where data can be skewed by the random effect of litter (e.g., fetal weight, placental weight), a mixed model was used to compare groups where condition (e.g., maternal genotype) was a *fixed* effect and litter was a *random* effect. Except for the NKG2A blockade experiment, litters containing 3 or less fetuses were excluded from all analyses.

#### GWAS meta-analysis –

Meta-analyzed as well as individual-level GWAS data were generated by the InterPregGen Consortium (Morgan et al., 2014) and are deposited in the European Genome-phenome Archive (https://ega-archive.org). Accession numbers of the meta-analyses we accessed are: European cases and controls EGAD00010001984 (7219 cases, 155,660 controls); Central Asian cases and controls EGAD00010001985 (2,296 cases and 2059 controls); and the combined European/Central Asian cohorts EGAD00010001988 (9,515 pre-eclamptic mothers and 157,719 controls). A full description of these cohorts and their meta-analysis, using the fixed-effects inverse-variance method based on effect estimates and standard errors is described in Steinthorsdottir et al. (2020).

Figures 2D, 2E, 2F, 2G, 2I, 3C, 4A, 5A, S3D-E, S4A and the graphical abstract were created in or with additional imaging from biorender.com.

Figure 5B was created in metascape.com.

## Supplemental information

Document S1. Figures S1–S5 and Tables S1 and S2

Data S1. List of processed gene expression scores, related to Figure 5

Video Abstract

Document S2. Article plus supplemental information
