# Supplementary material for: The CD94/NKG2A inhibitory receptor educates uterine NK cells to optimize pregnancy outcomes in humans and mice
Source: Immunity. 2021 Jun 8;54(6):1231–1244.e4. doi: 10.1016/j.immuni.2021.03.021 (PMC8211638; doi:10.1016/j.immuni.2021.03.021)
Supplement: Document S1. Figures S1–S5 and Tables S1 and S2 [file mmc1.pdf]

Immunity, Volume *54*

Supplemental information

The CD94/NKG2A inhibitory receptor educates uterine NK cells to optimize pregnancy outcomes in humans and mice

Norman Shreeve, Delphine Depierreux, Delia Hawkes, James A. Traherne, Ulla Sovio, Oisin Huhn, Jyothi Jayaraman, Amir Horowitz, Hormas Ghadially, John R.B. Perry, Ashley Moffett, John G. Sled, Andrew M. Sharkey, and Francesco Colucci

Supplemental Figure 1 related to Figure 1

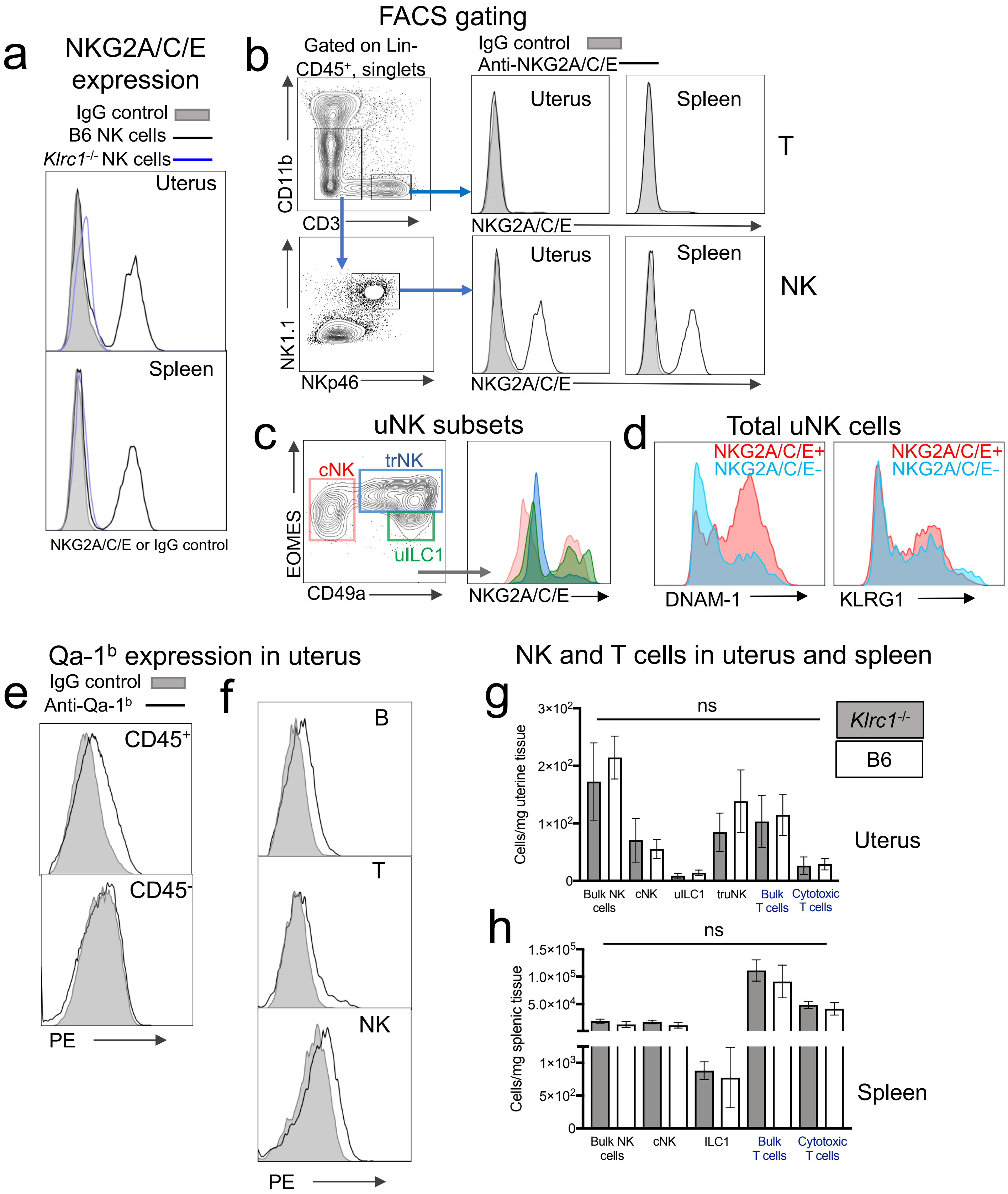

Supplemental Figure S1 related to Figure 1. NKG2A expression, FACS gating strategy and lymphocyte populations in *Klrc1*$^{-/-}$ mice.

a) Flow cytometric analysis showing NKG2A expression on uNK and spleen NK cells in B6 dams but not in *Klrc1*$^{-/-}$ NKG2A deficient dams (blue), both E10.5. Note that the antibody can also detect NKG2C and NKG2E, which are not detected in NKG2A deficient mice, showing that NKG2C and NKG2E, as reported before, are not expressed in mice of B6 background. **b)** Representative flow cytometric gating showing negligible expression of NKG2A on T cells and bimodal expression of NKG2A in NK cells in the uterus and spleen of B6 dams (both E10.5, 2-3 experimental repeats). **c)** NKG2A bimodal expression on tissue-resident uNK (trNK), conventional (cNK) and uterine ILC1 (uILC1). **d)** Representative (from 2 experimental repeats) flow cytometric profiles of DNAM-1 and KLRG1 on uNK cells. **e)** Flow cytometric analysis showing expression of the NKG2A ligand Qa-1^{b} on CD45^{+} leucocytes but not on CD45^{-} stromal cells from uterus; **(f)** Qa-1^{b} in uterine B, T and NK cells in B6 dams. (**g, h**) Absolute NK and T cell numbers/mg in the uterus and spleen in B6 (white bars) and *Klrc1*$^{-/-}$ (grey bars) dams at E10.5 (n= 4-5 dams per group, both mated with B6 males). All error bars represent standard deviation.

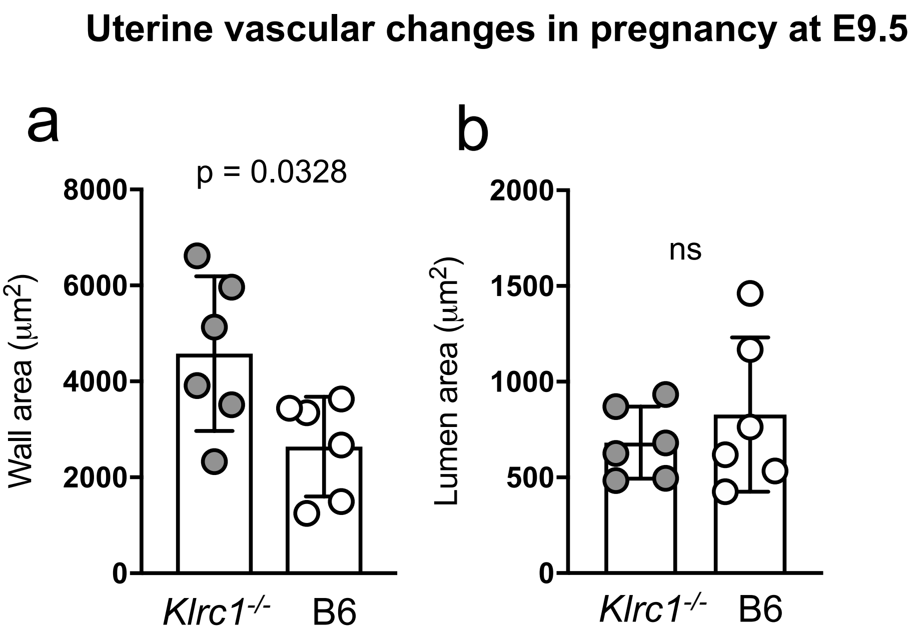
Uterine vascular changes in pregnancy at E9.5
a
p = 0.0328
Wall area (μm2)
8000
6000
4000
2000
0
Klrc1-/-
B6
b
ns
Lumen area (μm2)
2000
1500
1000
500
0
Klrc1-/-
B6

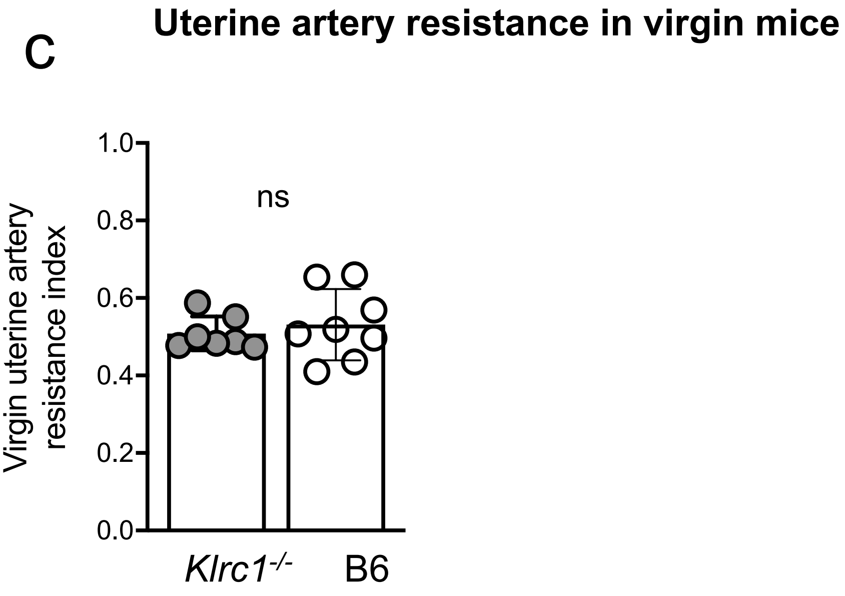
Uterine artery resistance in virgin mice
c
ns
Virgin uterine artery resistance index
1.0
0.8
0.6
0.4
0.2
0.0
Klrc1-/-
B6

Supplemental Figure S2 related to Figure 2. Uterine vascular changes in pregnancy and uterine artery resistance in virgin mice.

a) Wall area and lumen area **(b)** of E9.5 spiral arteries, by maternal strain (n=2-4 mice, each data point represents the mean of 15 analysed arteries in one implantation site, t-test). **c)** Uterine artery resistance index (UARI) comparison in virgin *Klrc1*$^{-/-}$ and B6 female mice. Each data point represents one mouse, t-test. All error bars represent standard deviation.

Supplemental Figure 3 related to Figure 3

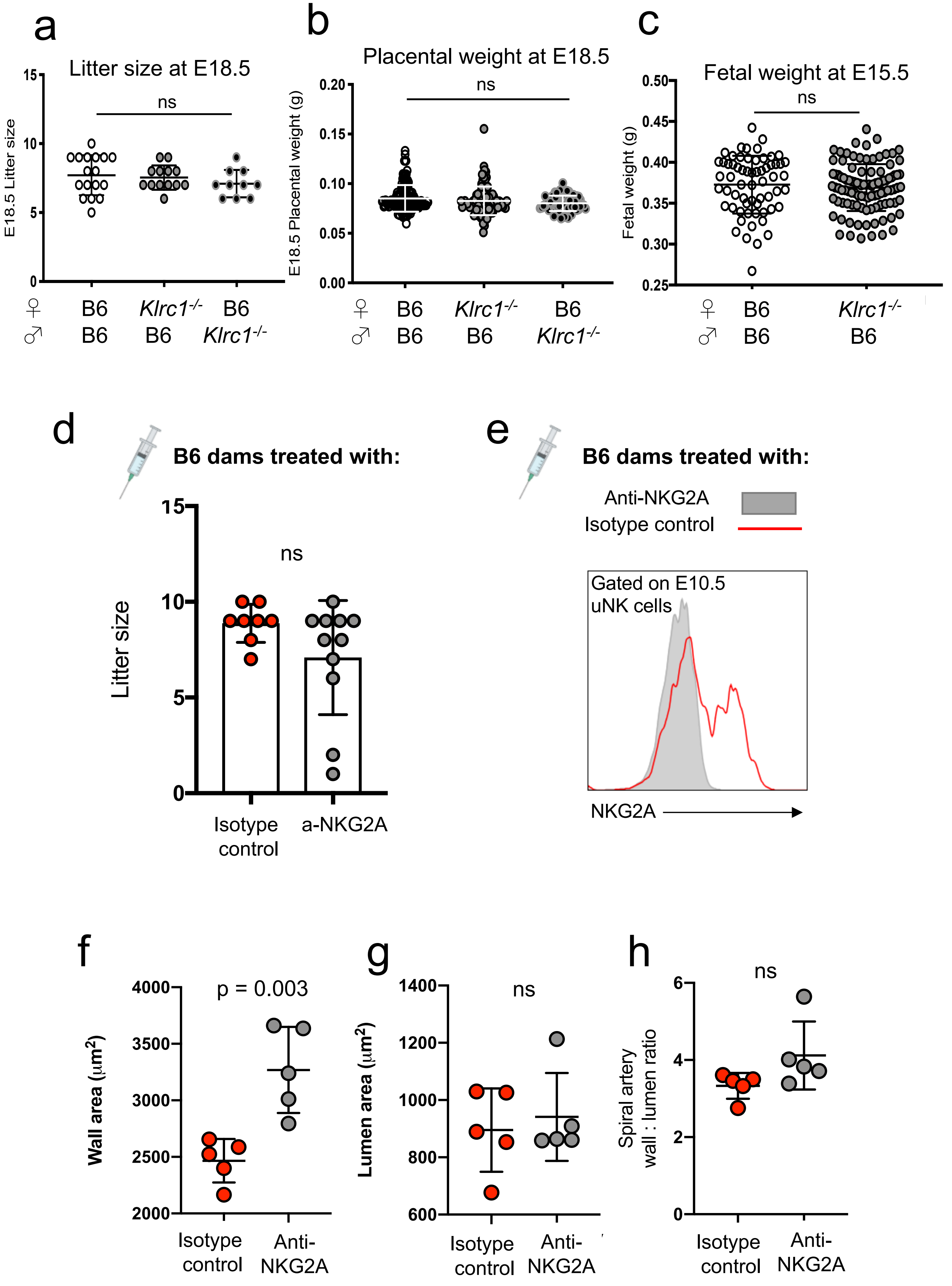

Supplemental Figure S3 related to Figure 3. Litter size and feto-placental weight in *Klrc1*$^{-/-}$ dams and in B6 dams treated with blocking anti-NKG2A antibody.

a) Each data point represents one litter. **b)** Each data point represents one individual placenta. **c)** Each data point represents one fetus, mixed model. **d)** Representative (from 2 experimental repeats) histogram of NKG2A staining on uNK at E10.5 after either isotype (red) or antibody injection (grey) at E6.5 and E9.5. **e)** Comparison of litter size at E18.5, after treatment of either isotype matched control antibody (red) or anti-NKG2A (grey) at E6.5 and E9.5 (Mann-Whitney U test, each datapoint represents one litter). **f)** Blocking anti-NKG2A antibody treatment causes increased vessel-wall area but no significant changes in lumen area **(g)** nor in wall:lumen ratios **(h)** of spiral arteries as assessed by stereology in B6 dams (E9.5) compared with B6 dams (E9.5) treated with isotype-matched control. Dams injected at E6.5. Each datapoint represents the mean of 15 measurements in one implantation site (t-test). All error bars represent standard deviation. Please also see data in Table 2.

Supplemental Figure 4 related to Figure 4

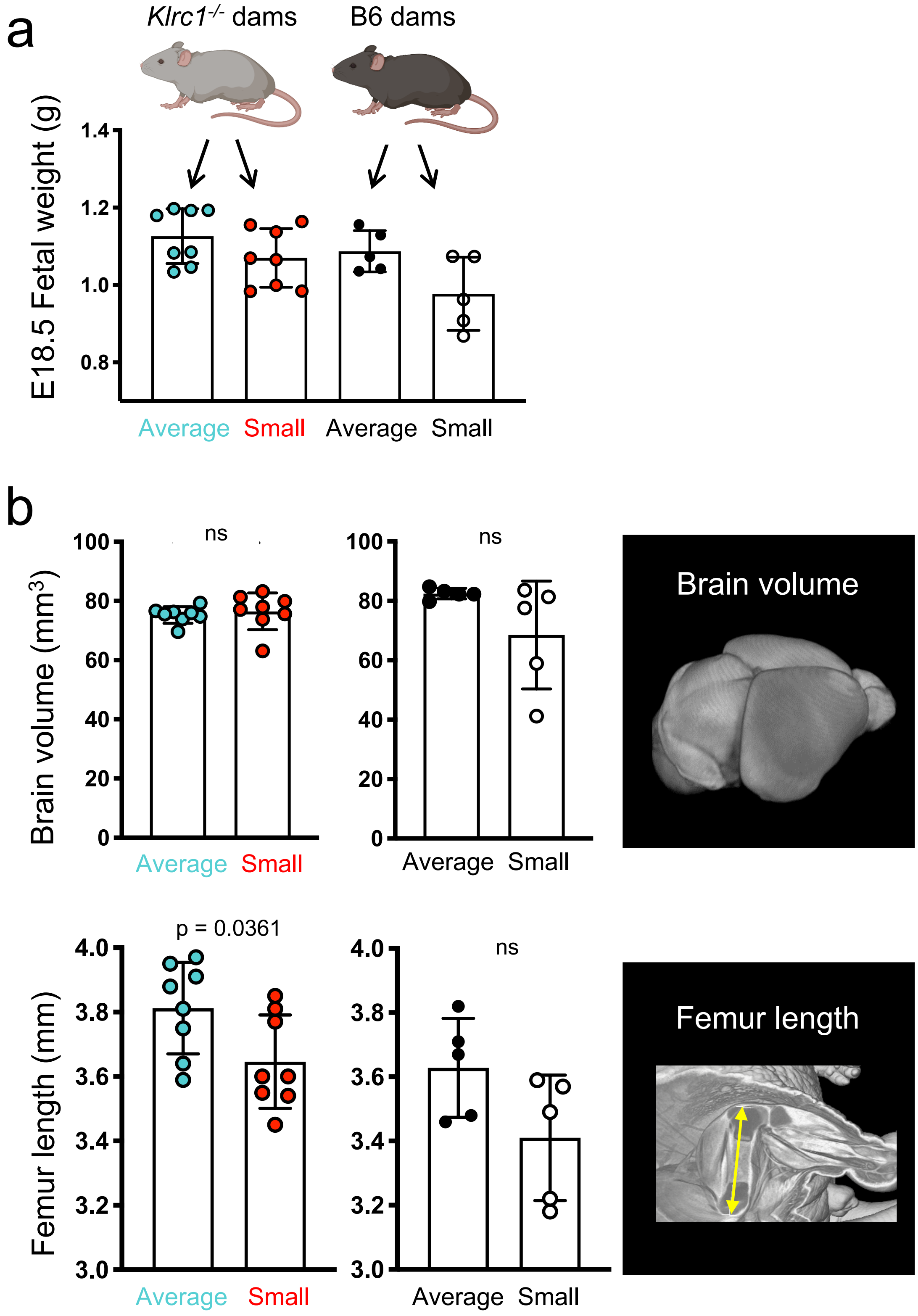

Supplemental Figure S4 related to Figure 4. Asymmetric growth in small fetuses from *Klrc1*$^{-/-}$ but not from B6 dams.

(a) Weight of all 26 selected E18.5 fetuses representing average-weight fetuses and small fetuses from either *Klrc1*$^{-/-}$ or B6 dams. (b) Comparisons of brain volume (top panel) and femur length (bottom panel) as measured by Micro-CT (illustrated in the panel on the right) between average-weight fetuses and small fetuses (each data point represents one fetus, n = 4-5 litters, t-test). All error bars represent standard deviation.

Supplemental Figure 5 related to Figure 5

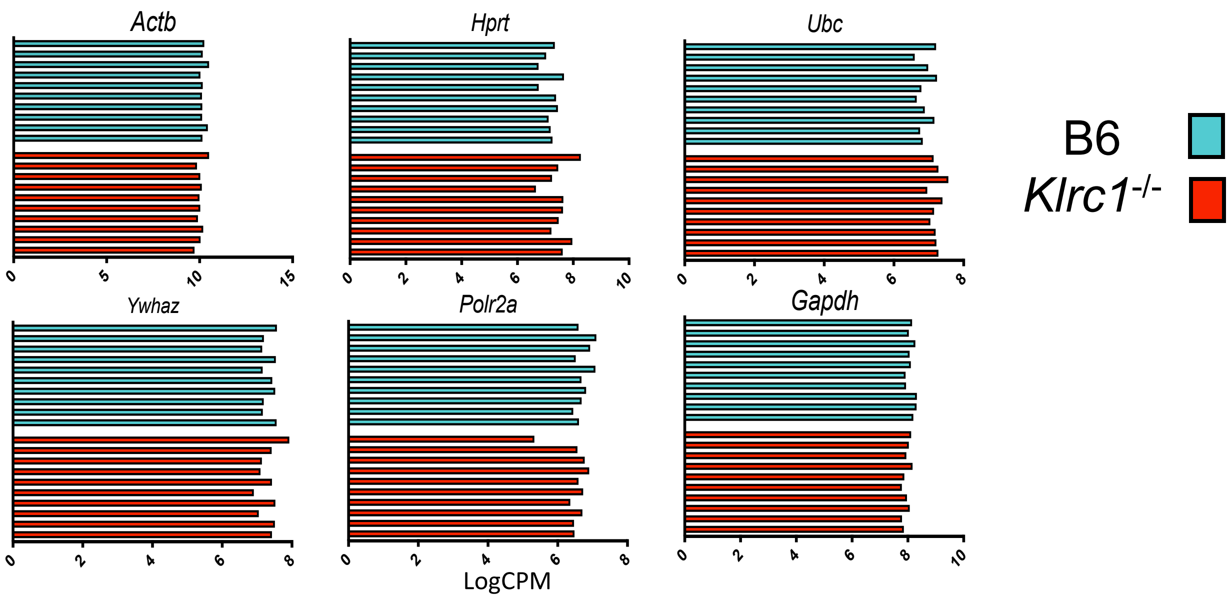

Supplemental Figure S5 related to Figure 5. Stable expression of placental housekeeping genes for each sample, by group.

Supplemental Table S1 related to Figure 5 and Figure S5

Ensembl ID	Gene Name	logFC*	logCPM	P Value	FDR
ENSMUSG00000063787	*Chchd1*	0.37	5.19	4.49E-07	0.0026
ENSMUSG00000057615	*Ldoc1*	-0.53	4.75	4.96E-07	0.0026
ENSMUSG00000037361	*Sf3b6*	0.40	6.03	9.64E-07	0.0033
ENSMUSG00000026192	*Atic*	-0.36	5.09	2.65E-06	0.0065
ENSMUSG00000039016	*Timm8b*	0.39	5.03	3.07E-06	0.0065
ENSMUSG00000028936	*Rpl22*	0.46	6.61	4.35E-06	0.0077
ENSMUSG00000078713	*Tomm5*	0.36	4.93	6.26E-06	0.0079
ENSMUSG00000090733	*Rps27*	0.45	8.21	6.55E-06	0.0079
ENSMUSG00000087687	*Pet100*	0.48	4.39	6.73E-06	0.0079
ENSMUSG00000052033	*Pfdn4*	0.50	4.24	7.59E-06	0.0080
ENSMUSG00000039221	*Rpl22l1*	0.33	6.75	4.61E-05	0.0433
ENSMUSG00000061477	*Rps7*	0.34	7.81	4.92E-05	0.0433
ENSMUSG00000071528	*Usmg5*	0.38	5.36	6.04E-05	0.0435
ENSMUSG00000022450	*Ndufa6*	0.30	5.57	6.32E-05	0.0435
ENSMUSG00000007209	*Ceacam9*	-0.71	2.99	6.36E-05	0.0435
ENSMUSG00000061787	*Rps17*	0.32	7.37	6.59E-05	0.0435
ENSMUSG00000020460	*Rps27a*	0.38	8.26	7.10E-05	0.0441
ENSMUSG00000043110	*Lrrn4*	-0.76	2.96	7.95E-05	0.0467
ENSMUSG00000020056	*Washc3*	0.34	5.64	8.81E-05	0.0490

Table S1. Differentially expressed placental genes from *Klcr1*$^{-/-}$ and B6 dams

***logFC** indicates the log (in base 2) fold difference between the two groups, where a negative value (underlined) represents a relative decrease in expression in the placentae of fetuses carried by *Klrc1*$^{-/-}$ dams as compared to those carried by B6 dams. **logCPM** (counts per million) is the log-average abundance across samples. P-value is for the differential expression test. FDR (false discovery rate) is the adjusted p-value for multiple testing.
